# The Medical News Pocket Formulary

**Published:** 1906-02

**Authors:** 


The Medical News Pocket Formulary. By E. Quin Thornton, M.D., Assistant Professor of Materia Medica in the Jefferson Medical College, Philadelphia. New edition. Leather, wallet shape for the pocket. $1.50 net. Lea Brothers & Co., Philadelphia and New York.

Professor Thornton has again brought this useful pocket companion thoroughly to date. It is arranged alphabetically and the best therapeutics of the masters is therefore instantly at command in its pages. A peculiar and valuable feature is found in the discriminating annotations. The various stages and complications of disease require treatment adapted to the conditions they present, and rational and successful therapeusis must recognize this fact. In a few clear words Professor Thornton points out the special utility of each of the various prescriptions recommended under the headings of the respective diseases. The book opens with sixteen pages of data which the physician will find useful, such as incompatibles, poisons and antidotes and a table of maximum and minimum doses. With this handy volume in his pocket, the practitioner need never feel at a loss for a pertinent suggestion.

